# Genetic and Epigenetic Signatures in Human Hepatocellular Carcinoma: A Systematic Review

**DOI:** 10.2174/138920211795564359

**Published:** 2011-04

**Authors:** Naoshi Nishida, Ajay Goel

**Affiliations:** 1 Department of Gastroenterology and Hepatology, Kyoto University Graduate School of Medicine, 54 Kawahara-cho, Shogoin, Sakyo-ku, Kyoto 606-8507, Japan; 2 Division of Gastroenterology, Department of Internal Medicine and Charles A Sammons Cancer Center and Baylor Research Institute, Baylor University Medical Centre, Dallas, TX 75246, USA

**Keywords:** Oncogenic pathway, hepatocellular carcinoma, DNA methylation, mutation, oncogene, tumor suppressor gene.

## Abstract

Hepatocellular carcinoma (HCC) is the third most common cause of cancer deaths worldwide, and the incidence of this fatal disease is still on rise. The majority of HCCs emerge in the background of a chronic liver disease, such as chronic hepatitis and liver cirrhosis. The current understanding is that majority of HCCs evolve as a consequence of chronic inflammation and due to the presence of infection with hepatitis viruses. These underlying pathogenic stimuli subsequently induce a spectrum of genetic and epigenetic alterations in several cancer-related genes, which are involved in cell-cycle regulation, cell growth and adhesion. Such widespread genomic alterations cause disruption of normal cellular signaling and finally lead to the acquisition of a malignant phenotype in HCC. In general, the type of gene alterations, such as point mutations, deletion of chromosomal regions and abnormal methylation of gene promoters differ according to the individual targeted gene. In HCC, incidence of genetic alterations is relatively rare and is limited to a subset of few cancer-specific genes, such as the tumor suppressor *p53*, *RB* genes and oncogenes such as the *CTNNB1. *In contrast, epigenetic changes that involve aberrant methylation of genes and other post-transcriptional histone modifications occur far more frequently, and some of these epigenetic alterations are now being exploited for the development of molecular diagnostic signatures for HCC. In addition, recent findings of unique microRNA expression profiles also provide an evidence for the existence of novel mechanisms for gene expression regulation in HCC. In this review article, we will review the current state of knowledge on the activation of various oncogenic pathways and the inactivation of tumor suppressor pathways in HCC that result in the disruption of cancer-related gene function. In addition, we will specifically emphasize the clinical implication of some of these genetic and epigenetic alterations in the management of hepatocarcinogenesis.

## INTRODUCTION

It is now widely accepted that stepwise accumulation of mutations in cancer-related genes and chromosomal alterations are involved in human carcinogenesis [1]. However, in the last decade we have recognized that in addition to genetic artifacts, epigenetic alterations that involve aberrant methylation of gene promoters and dysregulated expression of microRNA (miRNA), constitute equally important mechanisms of genomic instability in human cancers. Studies of these molecular alterations in hepatocellular carcinoma (HCC) have also revealed that this malignancy involves a multipathway process, and accumulation of genetic and epigenetic events leads to an abnormal activation or inactivation of multiple cellular signaling pathways including cellular proliferation, cellular survival, differentiation, and angiogenesis. Additionally, the emerging consensus is that the core biological processes including regulation of p53/ARF, RB/INK4A and Wnt/β-catenin pathways are commonly affected in a majority of HCCs regardless of the etiologies, suggesting the presence of a common oncogenic process of HCC development. 

This review provides an overview of genetic and epigenetic alterations observed in HCC, and discusses how these abnormalities relate to the disruption of specific biological signaling for the maintenance of homeostasis in normal hepatocytes. It is anticipated that such precise analyses of genomic profiling will reveal a global scheme of molecular classification of HCC, aid in the development of novel molecular-targeted therapy for specific subclasses of HCC, as well as assist once in the discovery and development of diagnostic and predictive biomarkers of HCC.

## P53/ARF PATHWAY

The p53/ARF pathway plays a key role in a variety of cellular functions, such as regulation of cell cycle, apoptosis and DNA repair. Cellular stress activates this pathway by activating the p53 protein as a transcription factor, and leads to the induction of the transcriptional network of p53-responsive genes [2]. The p53 circuit communicates with several fundamental cellular signals, such as the Wnt/β-catenin, RB/INK4a and p38 MAP pathways. Because the p53 protein has a variety of important functions for the maintenance of cellular responses, disruption of the p53/ARF pathway has been reported in almost every type of cancer including hepatocellular carcinoma (HCC) [3]. Notwithstanding the existence of multiple auto-regulatory loops that control p53 activity, MDM2 protein plays a central role in this process. The ARF antagonizes functions of MDM2 to induce regulatory responses that depend on the activation of p53 and its target genes. Although disruption of the p53 occurs in a subset of HCC, more than half of tumors retain a wild-type p53, suggesting that alteration of other molecules involved in this pathway might also contribute to hepatocarcinogenesis [4].

### The *p53* Gene

The *p53* gene is located on 17p13.1 and encodes this transcription factor with a tumor suppressor function. In human HCC, common alterations of the *p53* gene are point mutations within the conserved region of exons accompanied by loss of the short arm of chromosome 17. Generally, 30 - 60% of HCC carry mutations of the *p53 *gene [3]. The spectrum of point mutations reported in HCC varies according to the etiology of the underlying molecular pathogenesis. For example, aflatoxin B1 (AFB1) reportedly induces G:C to T:A transversion mutation in codon 249 of the *p53 *[5, 6]. As this type of mutation is detected in serum of HCC patients with an exposure to AFB1, this can be applied for risk assessment of HCC, and as a potential biomarker of HCC emergence and exposure to AFB1 [7]. However, it has been shown that AFB1-related liver tumor in rats didn’t carry any specific mutations that corresponded to codon 249 of human HCC [8]. On the other hand, transgenic mice with the hepatitis B virus-X (HBx) gene, in concert with AFB1 intake, develop HCC harboring G:C to T:A transversion mutation at the site corresponding to the codon 249 of the human *p53 *[9]. This evidence highlights the potential scenario where both AFB1 insult and infection with HBV are required for the evolution of specific mutations within the *p53* gene in HCC.

On the other hand, even though the mutational spectrum of the *p53* gene in AFB1-negative HCC is heterogeneous, the majority of mutations are still confined to a conserved region of DNA-binding domain encoded on exons 5 – 9 [4]. 

Other reports suggest that viral proteins affect the function of the p53 protein and contribute to HCC formation. For example, HBx protein itself reportedly binds to p53 and disturbs its capacity for DNA binding, transcription and induction of apoptosis [10]. None of the previous reports have provided any evidence for the direct action of HCV-related protein to p53. However, it has been shown that, nitric oxide (NO), which is induced by inflammatory cytokines such as TNF-α a and IFN-γ in chronic hepatitis C virus (HCV) infection, can induce mutation of cancer-related genes including the *p53*. In cases of colon cancer and lung cancer, nitric oxide synthetase 2 activity in the tumor closely related to the G:C to A:T mutations in the *p53* gene within the CpG sites [11], which is the major type of mutation in the *p53 *gene found in HCC without AFB1 contamination. 

### The *CDKN2A* (*p14^ARF^*) Gene

The *CDKN2A* gene encodes two tumor suppressors, p14^ARF^ and p16^INK4a^, which share the same second exons. The p14^ARF^ transcript functions as a stabilizer of the p53 protein. The p14^ARF^ interacts with the MDM2 protein, which is responsible for the degradation of p53 and in regulating the control of cell cycle during G1-S phase [12]. This gene is frequently mutated or deleted in a wide variety of human tumors including HCC. Generally, 7% of HCC carry homozygous deletion of the *CDKN2A* gene, and inactivation of the p14^ARF^ gene *via *aberrant methylation its gene promoter is a frequent event in HCC [13]. However, according to our analysis, methylation of the promoter of p14^ARF^ was also evident in small proportion of non-cancerous liver tissues as well, but it was far more frequent in HCC tissues [14]. In addition, a decrease of mRNA level was detected exclusively in HCC with homozygous deletion of this gene. From this point of view, the role of methylation at the promoter of p14^ARF ^may still be viewed somewhat controversial in pathogenesis of HCC [14].

### The *CDKN1A* and *CDKA1B* Genes

Although alterations in the genomic sequences of the tumor suppressor *CDKN1A* and *CDKN1B* genes are rare in HCC, the down-regulation of these cell-cycle regulatory proteins has been frequently reported [15-17]. Expression of p21^WAF1^, the product of the *CDKN1A* gene, is regulated by the tumor suppressor p53, and p21^WAF1^ in turn regulates the activities of cyclin-dependent kinases (CDK) 2 and 4, which control the G1-S and G2-M phase of cell cycle, respectively. In human HCC, down-regulation of p21^WAF1 ^has been observed and it associates with tumor progression and bad prognosis of the disease [15]. The virus-related protein of HBx and core protein of HCV are also known to suppress transcription of p21^WAF1^ [16].

P27^kip1^, the product of the *CDKN1B* gene, is also a member of cyclin dependent kinase inhibitors (CDKI) and inhibits kinase activity of cyclin E-CDK2 and cyclin D-CDK4. The expression level of this CDKI was also correlated to the poor outcomes for disease-free survival of HCC cases [17]. The protein level of p27^kip1 ^was reportedly lower in cirrhotic liver of non-cancerous tissues of HCC cases than in those without HCC, and the down-regulation of its expression was associated with promoter methylation of the *CDKN1B* gene [17, 18].

## RB/INK4A PATHWAY

The RB/INK4A pathway plays a central role in the regulation of the G1-S phase of cell cycle progression. It is known that RB activities associate with its phosphorylation status, and phosphorylation of RB depends on cell cycle progression coupled with the activation of CDK. The unphosphlyrated RB binds to the E2F-1 transcription factor, preventing it from interacting with the transcriptional machinery within the cell. The phosphorylated RB sequesters E2Fs, which are responsible for the transcription of many genes [19]. Among these genes, cyclin E, which binds to the CDK2, induces DNA replication during cell cycle. The majority of tumors, including HCC, frequently harbor aberrations of the members of this pathway. Our analyses indicate that 81% of HCC showed alteration of at least one component of the RB/INK4A pathway [20-22]. 

### The *RB* Gene

Loss of RB activity has been identified in many types of cancers including retinoblastoma, osteosarcomas, small cell and non-small cell lung cancers, breast cancer and HCC. In human HCC, 18–48% of tumors represent chromosomal loss of 13q14 region, where the RB gene resides. Our previous reports demonstrate that some HCCs with loss of heterozygosity on 13q14 carry additional structural alterations of the interstitial deletion in the RB gene, suggesting that a double hit on this locus leads to complete functional inactivation of RB [23]. On the other hand, down-regulation of RB protein is observed in 30–50% of HCC tissues compared to their corresponding non-neoplastic liver tissues [24]. Although the precise mechanism of the down-regulation of RB has not been clarified completely, some reports have suggested that abnormal methylation of the RB promoter may cause transcriptional inactivation of the gene in cancer cells [25].

### The CDKN2A (p16^INK4a ^) Gene

As described previously, p16^INK4a^ is a tumor suppressor protein, which is encoded by the *CDKN2A* gene. This molecule plays an important role in regulating the cell cycle, by inhibiting CDK4 and controlling cell cycle progression at the G1 phase. Mutation of p16^INK4a^ is also involved in the development of a variety of cancers including HCC. 

**Fig. (1):**
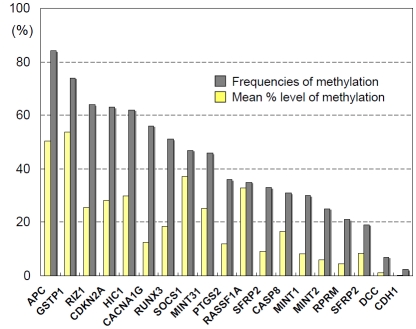
Mean methylation levels and frequencies of hypermethylation of several tumor suppressor genes (TSGs) in HCC. In contrast to genetic alterations such as point mutation, a variety of TSGs are frequently inactivated *via* epigenetic mechanism, suggesting that DNA methylation is a major alteration as a driver for human hepatocarcinogenesis.

Some HCCs carry homozygous deletions or point mutations of p16^INK4a^ [26]. However, recent data suggests that the major mechanism of inactivation of this gene is abnormal methylation of its gene promoter. As much as 40-70% of HCCs demonstrate p16^INK4a^ methylation, and this aberrant methylation associates with the down-regulation of the protein expression [27]. Since low-levels of methylation were also detected in the background non-cancerous liver of the HCC patients, we normalized methylation levels in HCC tissues to the background aberrant methylation and determined that p16^INK4a^ methylation inactivates the gene in 63% of human HCC cases (Fig. **1**) [28]. Previous reports suggest that p16^INK4a^ methylation also associates with the presence of HBV or HCV infection [29]. 

As described above, there is a close association between RB/INK4A pathway and p53/ARF pathway, especially in the regulation of cell cycle progression and apoptosis. We summarized the association of the responsible molecules in these pathways and the type of their alterations found in HCC in Fig. (**2**).

## WNT/β-CATENIN PATHWAY

Activation of Wnt/β-catenin pathway facilitates the expression of several genes indispensable for cell growth, such as c-myc and cyclin D1, through translocation of β-catenin to nucleus and its interaction with various transcription factors. 

In general, β-catenin within cells is present in the form of complexes with several proteins, and the ability of β-catenin to bind to other proteins is regulated by tyrosine kinases and serine kinases, such as glycogen synthase kinase (GSK)-3β. The binding proteins include cell adhesion molecules, several transcription factors, and axin, which is a component of the Wnt signalling pathway. β-catenin is associated with the axin complex together with GSK-3β and APC (adenomatosis polyposis coli) [30, 31]. This complex substantially induces the increase in phosphorylation of β-catenin by facilitating the action of GSK-3β, which leads to ubiquitin-dependent degradation of β-catenin by proteosomes. The action of GSK-3β is inhibited by the binding of Wnt to its receptors, which leads to the translocation of β-catenin to the nucleus and induction of a variety of functions within this pathway [32]. β-catenin also acts in conjunction with the T-cell factor (TCF)/lymphoid enhancing factor (LEF) family transcription factors to activate several target genes. 

Among the components of the Wnt/β-catenin pathway, the *APC* gene is known to be the primary target in patients with familial adenomatous polyposis (FAP) colon cancers [33]. Similarly, mutation of the *AXIN2* is a cause of attenuated polyposis [34], and mutation of the *CDH1* gene is known to induce familial gastric cancer [35]. Alterations of several components of this pathway are also common events in human HCC (Fig. **3**).

### The *CTNNB1* Gene

β-catenin is encoded by the *CTNNB1* gene and plays an important role in various aspects of liver biology including pathogenesis of liver cancer. Mutations in this gene are the cause of several cancers, such as colorectal cancer and ovarian cancer [36, 37]. In human HCC, point mutations or deletions at the phosphorylation site on neighboring codons in the exon 3 of the *CTNNB1* have been proposed to cause deregulation of the signaling function and contribute towards HCC pathogenesis. Generally speaking, 10–30 % of HCC carries mutations of the *CTNNB1* gene, which induces the accumulation of β-catenin in the nucleus [28]. 

### The *AXIN* Genes

As described above, the axin protein interacts with APC, β-catenin, GSK-3β, and protein phosphate 2. Mutations in this gene are also associated with hepatocellular carcinoma, hepatoblastomas, and several other cancers. Mutations of the *AXIN1* gene can be detected in 5–9 %, and mutations of the *AXIN2* are reported in ~3% of human HCC or HCC cell lines that carry the wild type *CTNNB1* gene [38]. Gene transfer of wild-type *AXIN1* resulted in enhanced apoptosis in HCC cell lines as a consequence of increased accumulation of β-catenin in the nucleus. On the other hand, some target genes of β-catenin, such as glutamine synthetase, did not show an enhanced expression even in cell lines with the mutated *AXIN* genes, suggesting that the tumor suppressor function of this protein might act *via *additional pathways other than Wnt/β-catenin [39].

### The *APC* Gene

APC also forms complexes with axin and GSK-3β , and contributes in controlling cellular β-catenin levels through ubiquitin proteasome-dependent degradation of β-catenin. In human HCC, biallelic inactivation of the *APC* gene (mutation of the gene accompanied by loss of chromosomal region of the APC locus) was reported in a HCC case [40]. In our analysis, 84% of HCC carried dense methylation on the promoter of this gene in HCC (Fig. **1**), indicating that promoter methylation was the major mechanism of inactivation of the *APC* gene in human HCC [28].

### The CDH1 (E-Cadherin) Gene

E-cadherin is a calcium-dependent cell-cell adhesion glycoprotein that plays an important role in the formation of the epithelial cell layer. Cadherin can be found in complexes with catenin, which is important for the biological function of this gene. The oncogenic signals from EGF and Src also target β-catenin. It has been shown that phosphorylation of β-catenin through the EGF pathway associated with invasion and metastasis of cancer because of suppression of the adhesion function of Cadherin [41]. Mutations of this gene are reported in gastric, colorectal and breast cancers. Loss of function of this gene contributes to the progression of HCC by permitting increased proliferation, invasion, and metastasis of the neoplastic cells. Expression analysis of E-cadherin in hepatocellular carcinomas demonstrated that 56% of HCC showed the down-regulation of E-cadherin expression, and this phenomenon directly correlated with the size of tumors, as well as the mitotic index and survival [42]. 

Several reports suggested that the *CDH1* gene can also be inactivated through the promoter hypermethylation of its promoter region. Interestingly, it has also been reported that reactive oxygen species (ROS) induce hypermethylation of the *CDH1*promoter. In this context, activation of PI3K/Akt/GSK-3β by ROS has been shown to recruitment of histone deacethylase and DNA methyltransferase on its promoter, which causes the downregulation of *CDH1*expression [43]. Because longstanding chronic inflammation enhances the production of ROS in the background liver of HCC, it is reasonable to speculate that these epimutations play an important role in the initial steps of inflammation-related hepatocarcinogenesis.

### Secreted *Frizzled*-Related Protein (*SFRP*) 2 

Frizzled are membrane proteins which are known as receptors of Wnt. SFRPs act as soluble modulators of Wnt signaling as SFRPs containing the putative Wnt-binding site of frizzled proteins. Because SFRPs are able to down-regulate Wnt signaling by forming an inhibitory complex with the frizzled receptors, SFRPs are considered as tumor suppressors. We have reported that one of the SFRP family genes, SFRP2, showed hypermethylation of its promoter and was frequently inactivated in human HCC (Fig. **1**) [28].

## ABNORMAL METHYLATION OF TUMOR SUPPRESSOR GENES

It is now increasingly being recognized that the patterns of DNA methylation, which are ordinarily maintained during normal cell division, are often disturbed in many human tumors, including HCC. Since low levels of DNA methylation can also be detected in the non-cancerous liver of patients with chronic liver damage or HCC, it is now believed that this epigenetic defect emerges at an early stage during hepatocarcinogenesis [44]. In human HCC, both regional hypermethylation of tumor suppressor gene (TSG) promoters and hypomethylation of repetitive DNA sequences are commonly observed [45]. The former is an important mechanism for the inactivation of corresponding gene, while the latter plays a role in maintaining chromosomal fragility and activation of retrotransposons and microRNA (miRNA), both of which could be critical for HCC development and progression. Although the precise mechanisms for the alteration of DNA methylation during hepatocarcinogenesis are still unclear, both HBx and HCV core proteins have been shown to induce the expression of DNA methyltransferases, which then leads to abnormal methylation of the *CDH1* gene [46, 47]. 

We have previously reported that DNA methylation of TSGs was closely associated with the presence of HCV not only in HCCs but also in chronic hepatitis tissues, suggesting that the chronic inflammation or presence of HCV may contribute to the emergence of abnormal methylation [48]. The numbers and frequencies of genetic alterations of cancer-related genes in HCC are limited, however, epigenetic alterations are frequently observed in several TSGs (Fig. **1**) [49].

## CLINICAL IMPLICATIONS OF GENETIC AND EPIGENETIC ALTERATIONS

Cancer specific alterations of several genes are promising candidates for developing molecular markers of HCC, such as diagnosis and prediction of prognosis. Among them, alteration of DNA methylation is widely and frequently observed in almost every HCC, and DNA methylation can be easily and quantitatively measured in a variety of clinical specimens. Detection of methylation of the *CDKN2A* and *GSTP1* genes in serum has been used for the early diagnosis of HCC [50, 51]. Furthermore, detection of the methylated *CDKN2A* and *RASSF1A* genes in serum of cases at a high risk of HCC could predict early occurrence of HCC [52]. Similarly, alteration of certain cancer-related genes or oncogenic pathways could be a target of developing a novel therapy of HCC. For example, a high level of copy-number gain and overexpression of vascular endothelial growth factor A was reported in a subset of HCC, suggesting that antiangiogenic therapies could be exclusively effective for this type of tumor [53].

## ALTERED EXPRESSION OF miRNAs

Expression of miRNAs, which has been identified as a new class of small non-coding family of genes that are involved in post-transcriptional gene regulation, is also shown to be frequently altered in HCC. Several comprehensive analyses of miRNA have identified differentially expressed miRNAs in various subsets of HCCs. This type of alteration can also modulate several important signaling pathways, which is critical for hepatocarcinogenesis. 

In human HCCs, miRNA (miR)-122 expression is significantly reduced in a subset of HCC compared to the non-cancerous tissues [54]. miR-122 can modulate cyclin G1 expression in HCC-derived cell lines, and an inverse correlation between miR-122 and cyclin G1 expression has been shown in human HCC, suggesting that cyclin G1 is a target of miR-122 [55]. On the other hand, miR-221 also targets cell-cycle inhibitors CDKN1B (p27^kip1^) and CDKN1C (p57^kip2^) and its altered expression also results in a disturbed normal cell-cycle regulation in hepatocytes [56]. 

MiR-21 is shown to be overexpressed in HCC as well as HCC cell lines, and inhibition of miR-21 increases expression of the tumor suppressor *PTEN *which helps in decreased tumor proliferation and invasion [57]. On the other hand, Ji, *et al. *showed that tumors with reduced miR-26 expression had a distinct transcriptomic pattern, with the activation of NF-κB and IL-6 signaling pathways [58]. Interestingly, induction of IL-6 is reported as an important step for inflammation-related HCC pathogenesis [59], and high levels of serum IL-6 can be a risk factor for HCC in HCV-positive patients [60]. Activation of this pathway also accounts for the gender differences and risk of HCC, as estrogen is known to suppress MyD88-dependent IL-6 production, and miR-26 is expressed at a higher level in women than men in the liver [58, 59]. Reportedly, this miRNA is also a good candidate for therapeutic target of HCC. Patients whose tumors had low miR-26 expression had shorter survival but a better response to interferon therapy than did patients whose tumors had high expression of this miRNA [58]. In addition, Kota, *et al. *reported the significance of this miRNA on treatment response using a mouse model [61]. Expression of miR-26 in liver cancer cells *in vitro* induces cell cycle arrest *via *suppression of cyclin D2 and cyclin E2, and systemic administration of miR-26 in mouse HCC model induces inhibition of cancer cell proliferation and induction of tumor-specific apoptosis without toxicity [61]. However, as individual miRNAs regulate hundreds of transcripts, antiproliferative effects of miR-26 in HCC, might not attribute to a single oncogeneic pathway but a regulation of multiple pathways, such as c-Myc and p53-dependent pathway.

On the other hand, several other studies revealed the association between expression profile of miRNA and etiology as well as specific genetic alterations. The miR-96 is overexpressed in HBV-related tumors, and miR-126 is down-regulated in alcohol-related hepatocellular carcinoma. Similarly, several reports suggest that the expression profile of miRNA can be applied to evaluate the etiology of tumor and predict tumor behavior, such as metastasis and prognosis [58, 62-66]. These evidences support the idea that in addition to genetic and epigenetic alteration of oncogenes and TSGs, the altered expression of miRNAs is an additional mechanism critical for human hepatocarcinogenesis. This concept is bound to receive a lot of attention in the coming years, as it not only helps in a better understanding of the molecular mechanisms of HCC, but also has a potential for clinical use including the development of biomarkers and therapeutic targets of this malignancy. 

## CONCLUSION

**Fig. (2):**
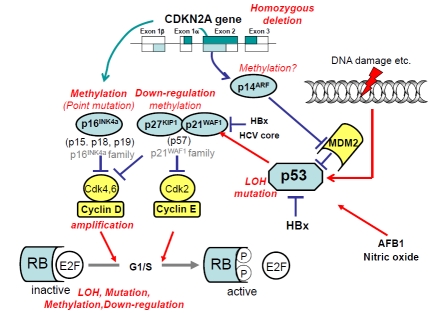
Schematic representation of alterations in p53/ARF and RB/INK4A pathways in HCC. Molecules with oncogenic function are shown in yellow and those with tumor suppressive function are shown in blue. Specific type of genetic and epigenetic alterations found in HCC are shown in red letter.

**Fig. (3):**
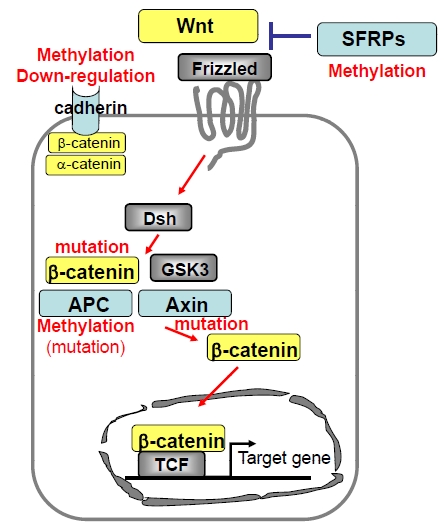
Schematic representation of Wnt/β-catenin pathway and the alterations reported in HCC. Functions of molecules shown in yellow and blue represent oncogenic and tumor suppressive respectively. Specific types of genetic and epigenetic alterations found in HCC are shown in red letter.

We have summarized in this review article the spectrum of various genetic and epigenetic alterations in a variety of genes and their corresponding oncogeneic pathways that are operational in human hepatocarcinogenesis (Figs. **2** and **3**). What we now understand is that similar to many other cancers, development of HCC associates with a step-wise accumulation of genetic and epigenetic defects in cancer-related genes that includes point mutations, chromosomal alterations, aberrant DNA methylation and alterations of miRNA expression. Although the precise underpinnings of these various processes are still hazy, nonetheless, it is very likely that the defects in multiple signaling pathways might overlap and crosstalk exists among these pathways, as the premalignant clones progress towards a more advanced stage of HCC. Given the high prevalence of chronic liver disease and HCC patients, knowledge of these molecular events is very exciting and timely, because an understanding of these events will help tailor approaches that can be best exploited for the early detection, management and therapy of HCC.
